# On the Biocompatibility and Teat Retention of In Situ Gelling Intramammary Formulations: Cattle Mastitis Prevention and Treatment

**DOI:** 10.3390/pharmaceutics13101732

**Published:** 2021-10-19

**Authors:** Sushila Bhattarai, Dhayaneethie Perumal, Michael J. Rathbone, Craig R. Bunt, Raid G. Alany

**Affiliations:** 1 School of Pharmacy, Faculty of Medical and Health Sciences, The University of Auckland, Auckland 1023, New Zealand; sushib23@hotmail.com; 2 Drug Discovery, Delivery and Patient Care (DDDPC) Theme, School of Life Sciences Pharmacy and Chemistry, Kingston University London, Kingston-upon-Thames KT1 2EE, UK; Dhayaneethie.Perumal@moe.gov.ae; 3 Commission for Academic Accreditation, MOE, Abu Dhabi P.O. Box 295, United Arab Emirates; 4 InterAg, Private Bag 3123, Hamilton 3204, New Zealand; michael.john.rathbone@gmail.com (M.J.R.); Craig.Bunt@lincoln.ac.nz (C.R.B.); 5 Department of Agricultural Sciences, Lincoln University, Lincoln 7647, New Zealand

**Keywords:** biocompatibility, in situ gelling intramammary formulations, cattle mastitis

## Abstract

Treatment and prevention of cattle mastitis remains a formidable challenge due to the anatomical and physiological constraints of the cow udder. In this study, we investigated polymeric excipients and solvents that can form, (when combined) novel, non-toxic and biocompatible in situ gelling formulations in the mammary gland of bovine cattle. We also report on a new approach to screen intramammary formulations using fresh excised cow teats. Fourteen hydrophilic polymers and six solvents were evaluated for in vitro cytotoxicity and biocompatibility towards cultured bovine mammary epithelial cells (MAC-T), microscopic and macroscopic examination upon contact with excised cow teats. No significant cytotoxicity (*p* > 0.05) was observed with polyethylene oxides, hydroxypropyl methylcellulose, carboxymethyl cellulose, sodium alginate and xanthan gum. Polycarbophil and carbopol polymers showed significantly higher cytotoxicity (*p* < 0.05). Concentration-dependent cytotoxicity was observed for glycerin, propylene glycol, polyethylene glycol 400, ethanol, N-methyl-2-pyrrolidone and 2-pyrrolidone, with the 2-pyrrolidone solvents showing higher cytotoxic effects (*p* < 0.05). In situ gelling formulations comprising hydroxypropyl methylcellulose or carboxymethyl cellulose and solvents in specific ratios were biocompatible at higher concentrations with MAC-T cells compared to alginates. All investigated formulations could undergo in situ sol-to-gel phase transformation, forming non-toxic gels with good biocompatibility in excised cow teats hence, showing potential for use as intramammary carriers for sustained drug delivery.

## 1. Introduction

Bovine mastitis continues to be one of the major challenges facing the dairy cow industry, causing heavy economic losses as a result of decreased milk production, reduced fertility, treatment costs or death in instances of therapeutic failure [1].

Mastitis is the inflammation of the mammary gland; it occurs primarily in response to intramammary bacterial infection in dairy cows amongst other aetiologies including fungal and algal infection, mechanical, thermal or chemical trauma [2,3]. The increased incidence of new infections is believed to occur mainly during the drying off or dry periods when dairy cows are particularly susceptible to mastitis due to predisposing factors.

Subclinical pathogens which have survived in the udder from the previous lactation may progress to manifest clinically after calving [4]. Furthermore, new infections caused by environmental pathogens, particularly the *Staphylococcal* strains including *Streptococcus uberis*, *Streptococcus dysgalactiae*, as well as *Corynebacterium bovis* or *E. coli*, are more frequent during the dry period. *Staphylococcus aureus* is the most common mastitis-causing pathogen [5,6].The causative pathogens involved in mammary gland infections present the greatest challenge in treating mastitis [7,8].

The pathogens isolated from mastitis-affected milk have been reported to exhibit a wide spectrum of antibiotic susceptibility. Therefore, antibiotics are often routinely administered to entire herds to treat mastitis, especially through intramammary infusion [6]. However, the wide use of antibiotics for the treatment of mastitis has meant the development of bacterial resistance to antibiotics. Several studies have assessed antibiotic sensitivity/resistance. For instance, in a study conducted in the Zenica region in Bosnia and Herzegovina, antimicrobial resistance was observed against benzylpenicillin (56.3%) and oxytetracycline (46.2%) [9]. Similarly, antibiotic resistance and virulence genes in the staphylococus strain was identified by Saidi et al. [6].

Since excessive use of antibiotics in dairy cows can contribute to increased antimicrobial resistance, further research must be directed towards the development of new therapeutic agents/techniques that can both replace conventional techniques and also solve the problem of emerging antibiotic resistance.

Some alternative approaches have concentrated on the treatment of mastitis by homeopathy [5] and the use of feed additives [10]. Other treatment strategies for the treatment/control of mastitis include dry cow therapy using external teat seals. Internal teat inserts and internal teat seals have also been developed and marketed to overcome the occurrence of mastitis.

The use of internal teat seals is indeed an effective strategy to prevent mastitis infection during the dry period. When infused into each quarter of the drying off udder, they behave in a similar way to a natural keratin plug in forming a barrier to environmental pathogens. They are intended for retention for weeks, hence decreasing new intramammary infections (IMIs) during the dry period [11,12]. Despite the success of teat seal products, inadequate and poor retention followed by subsequent failure to seal the teat was reported [13].

Furthermore, excipients in current formulations include heavy metal salts such as bismuth subnitrate [14,15,16,17,18] aluminum monostearate and mineral oils, for which there is information on their undesirable effect on cells and tissues that they contact. For example, bismuth subnitrate has been shown to induce neurotoxic effects in both humans and animals [19] with reported side effects being encephalopathy, nephropathy, stomatitis and colitis [20]. Moreover, some common vehicles are known to provoke inflammation and irritation to the bovine udder which may breach the integrity of the mucosa and predispose the udder quarters to infection [21].

To improve on some of the reported inadequacies of internal teat seals, we have reported on novel in situ gelling polymeric dispersions consisting of infusible low-viscosity blends of synthetic, semi-synthetic and naturally occurring polymers dispersed in different organic solvents, which undergo a rapid sol to gel phase change induced by solvent exchange (organic solvent–water) with the surrounding environment [22]. The system, which transforms into a semi-solid mass at the administration site upon hydration of the dispersed polymer, can assume the shape of the cavity where it is infused, forming a barrier against microbial adhesion or pathogen ingress [23]. Furthermore, these systems may serve as matrices for controlled delivery of drugs and bioactive compounds [22]. The phase behavior of various pseudoternary polymer/solvent blend/water systems, was investigated and six in situ gelling formulations were identified as promising for intramammary applications based on their rheological and mechanical properties.

These in situ gelling delivery systems are designed to come into intimate contact with biological tissues over extended periods of time (weeks to months), hence the significance of biocompatibility/cytotoxicity studies. The biocompatibility requirements for intramammary systems are such that they should demonstrate the absence of a cytotoxic effect [24] as well as biocompatibility and functionality, which contributes to the intended purpose of the system [25]. In addition, the system must be easily sterilizable during manufacture [24]. For this purpose, the International Organization for Standardization (ISO) guideline 10,993 specifies a series of standards for the evaluation of biocompatibility; it prioritizes cell culture-based in vitro tests to precede clinical testing.

More recently, we reported on antibiotic-free solid polymeric inserts for the prevention and/or treatment of bovine mastitis. Polyethylene oxide (PEO)-based inserts were prepared using different concentrations of various hydrophilic polymers and water-soluble and water-insoluble drug-release-modifying excipients [26].

This manuscript reports on the cytotoxicity and biocompatibility of hydrophilic polymers, solvents and intramammary formulations [22,26] on bovine mammary epithelial cells (MAC–T cells). Furthermore, a new method to screen intramammary formulations for potential in situ gelling using freshly collected excised cow teats is described.

## 2. Materials and Methods

### 2.1. Materials

Dulbecco’s modified Eagle’s medium containing high glucose, L-glutamine, sodium pyruvate, pyridoxine hydroxide, 0.25% Trypsin-Ethylenediamine-tetra acetic acid (EDTA), penicillin G (5000 units/mL) and streptomycin sulfate (5000 μg/mL) was from Gibco BRL (Carlsbad, CA, USA). Triton X-100 for cell lysis was from BDH chemicals (Poole, Dorset, UK). Solvents, propylene glycol (PG), glycerol (G), polyethylene glycol 400 (PEG), N-methyl 2-pyrrolidone (NMP) and 2-pyrrolidone (2-Pyr) were purchased from Sigma chemicals (St. Louis, MO, USA); ethanol from Scharlau Chemie S.A., (Barcelona, Spain); polymers, polyethylene oxide (PEO, Polyox^TM^, Bellevue, WA, USA) of different molecular weights (MW 100 K, 1 M, 4 M and 7 M) from Dow Chemical (Midland, MI, USA); hydroxypropyl methylcellulose (HPMC, Metolose 60 SH) from Shin-Etsu Chemical Co. Ltd., (Tokyo, Japan); sodium alginate (ALG) and high-viscosity grade carboxymethyl cellulose sodium (CMC) from BDH (Poole, Dorset, UK); xanthan gum (XG) from Jungbunzlauer GmbH, (Basel, Switzerland); polycarbophil (PCP), polyvinyl pyrrolidone (PV) and carbopols (CP: 974P, 907 and 971P) from BF Goodrich (Cleveland, UK). Bovine mammary epithelial cells (MAC-T) were kindly provided by AgResearch, Hamilton, New Zealand.

### 2.2. Preparation of Polymer and Solvent Systems

Polymers, polyethylene oxide (PEO, Polyox^TM^) of different molecular weights 100,000, 1 M, 4 M and 7 M, hydroxypropyl methylcellulose (HPMC), sodium alginate (ALG), carboxymethyl cellulose sodium (CMC), xanthan gum (XG), polyvinylpyrrolidone (PVP), carbopols (974P, 907 and 971P) and polycarbophil (PCP) were prepared/dissolved in culture media at a concentration of 10 mg/mL and left at 37 °C for 24 h to equilibrate. The samples were further diluted with culture media to concentrations ranging from 0.05 to 1 mg/mL.

Solvents, glycerine (G), propylene glycol (PG), polyethylene glycol 400 (PEG), ethanol (E), N-methyl 2-pyrrolidone (NMP) and 2-pyrrolidone (2-Pyr), were diluted to concentrations ranging from 0.1 to 10% *v/v* with culture media. The solutions of both polymers and solvents were prepared under aseptic conditions and were neutralised, whenever necessary, to physiological pH.

### 2.3. Preparation of Polymer/Solvent Intramamary Formulations

Polymer/solvent blends were aseptically prepared according to the compositions shown in Table 1. Briefly, the binary solvents (G:E or G:PG) were mixed (according to the ratios shown in Table 1) in glass beakers using a Heidolph star blade mixer (Nicholas Watson Victor Ltd., Germany) at ~250 rpm speed. Thereafter, 10 g polymer (HPMC, CMC or ALG) was slowly added while the mixture was stirred vigorously until polymer particles were wetted and disaggregated. Finally, water was added under stirring until a uniform suspension was produced. Test samples for cell culture studies were prepared by diluting each formulation blend with the culture medium to desired concentrations. The solutions of the various blends were neutralised, whenever necessary, to physiological pH.

### 2.4. Cell Culture

MAC-T cells (passage number 8) were cultured in 75 cm^2^ tissue culture flasks (Nunc, Roskilde, Denmark) and maintained in DMEM supplemented with 10% fetal calf serum (FCS) (Invitrogen, Inchinnan, UK), penicillin G and streptomycin (100 IU/mL), 0.5% insulin and 0.2% hydrocortisone. The cells were incubated at 37 °C in 5% CO_2_ until confluent at a density of 0.1 × 10^5^. Adherent cells were harvested using 0.25% Trypsin-0.05 mM EDTA. Growth curves of cells were established, and the assays were performed in the exponential growth phase.

In addition, cells were checked routinely by visual examination under an inverted phase-contrast microscope (CKX53 Olympus, Tokyo, Japan) to monitor changes in morphology and detachment of cells.

### 2.5. Celll Viability (MTT or MTS) Assay

The viability of MAC-T cells following exposure to test solutions of solvents, polymers or polymer/solvent blends for 72 h was quantitatively measured using an MTT assay.

MAC-T cells were seeded onto 96-well plates (Corning Incorporated, NY, USA) at a density of 3 × 10^3^ cell/cm^2^. After 24 h, the media was discarded from the well and the cells were washed with phosphate buffer saline (PBS). One hundred (100) µL of each test solution (polymer, solvent or polymer/solvent blend prepared in culture media with 1% FCS) was introduced into the well in triplicates and incubated for 72 h at 37 °C in 5% CO_2_. Untreated controls (cells without test solutions) and background controls (media only) were used as references.

After 72 h incubation, cell viability was quantitatively measured using 3-(4,5-dimethyl-2-yl)-5-(3-carboxymethoxyphenyl)-2-(4-sulfophenyl)-2H-tetrazolium (MTT or MTS) commercial assay EZ4U kit (Australian Laboratory Services) according to the manufacturer’s instructions. The EZ4U cell proliferation assay reagent (TECO medical Group, Sissach, Switzerland) (20 µL) was added to each well and the plates were further incubated for 4 h at 37 °C in 5% CO_2_. Colourimetric readings of cooled 96-well plates were undertaken at 490 nm using a Model-ELX 800 UV microplate reader (Bio-Tek, Instruments Inc, Winooski, VT, USA). The relative cell viability (%) was expressed as a percentage of the viability in untreated wells.

### 2.6. Cytotoxicity (LDH) Assay

The LDH assay was performed using a cytotoxicity detection kit (Roche Diagnostics, GmbH, Mannheim, Germany) according to the manufacturer’s protocol. Briefly, after incubation of MAC-T cells in 96-well plates overnight, media was discarded, cells washed with PBS and the test solution (100 µL/well) added to each well. After incubation at 37 °C in 5% CO_2_ for 72 h, plates were centrifuged at 250× *g* for 10 min and 50 µL of supernatant was withdrawn from each well and added to wells in a new plate. LDH reagent (50 µL) was added to each well. After 30 min incubation protected from light, absorbance values were measured at 490 nm with the microplate reader. Maximum LDH release was also measured using 1% Triton X-100 as high control (Hc) and cells without test substance were used as low control (Lc). Cytotoxicity was determined as a percentage of High control (Hc) after correcting for background absorbance.

### 2.7. In Situ Sol-to-Gel Formulation Transformation in Excised Bovine Teats

**Figure 1:**
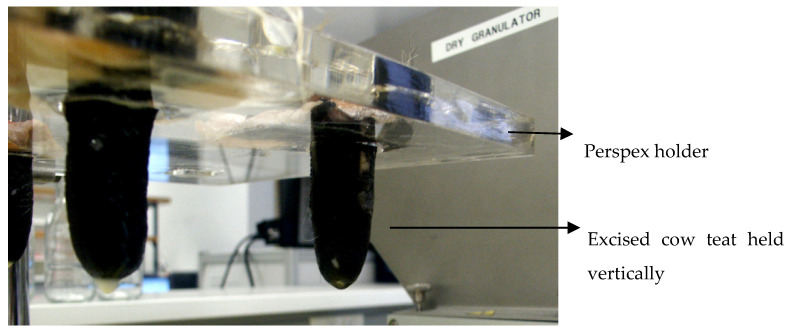
New ex vivo setup to study in situ gelling, retention and biocompatibility of formulations in excised bovine teats.

Teats were excised from the mammary glands of slaughtered cows and transferred immediately to our lab. They were mounted in a purpose-made frame maintained in a vertical position (Figure 1). Whole cow milk (2 mL) was introduced to each teat. Immediately thereafter, 3 mL of each polymer/solvent blend formulation was infused into the teat cistern via the streak canal and left for 3 and 12 h while shaking at 50 rpm at room temperature. Teats were cut open (at two time points of 3 and 12 h) and evaluated for evidence of in situ gelling of the formulations. In addition, the mucosal tissue in direct contact with the formulation was inspected for any undesirable events (redness, inflammation, pallor and necrosis). Each polymer blend (HPMC 1, HPMC 2, CMC 1, CMC 2, ALG 1 and ALG 2) was investigated in triplicate.

### 2.8. Statistical Analysis

Statistical analyses for any differences between test and control samples were carried out using one-way analysis of variance (ANOVA) (Minitab Release, version 12.1 for Windows, Harrisburg, PA, USA). A Tukey’s pair wise comparison set at a 95% confidence interval was performed.

## 3. Results

### 3.1. Morphology of Mac-T Cells

**Figure 2:**
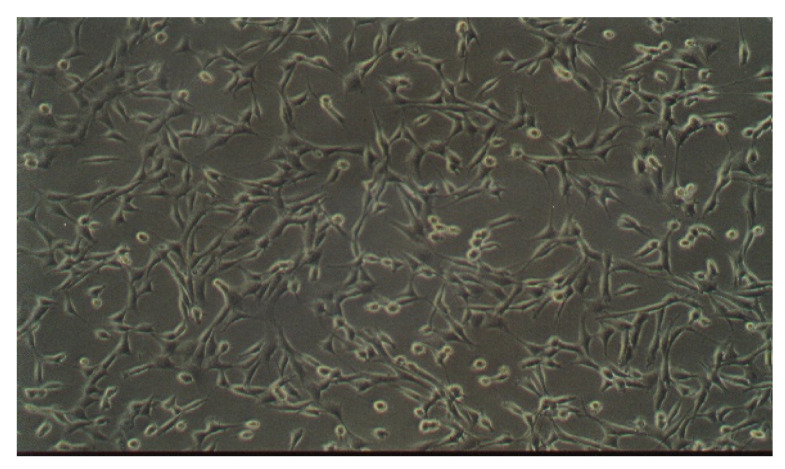
Light micrograph of MAC-T cells showing their characteristic morphology (100× magnification).

MAC-T cells (Figure 2) are produced from primary bovine mammary epithelial cells [27]. The cells retain a number of biochemical and morphological characteristics in vivo [27,28]. Huynh et al. reported that MAC-T cells possess the ability to differentiate and secrete caseins, providing unique mammary epithelial cell function [29]. Hence, MAC-T cells were selected as a mammary gland epithelium cell model for our biocompatibility studies.

Cell culture showed a characteristic cobblestone morphology of epithelial cells (Figure 2). Viable cells adhered to the surface of the flask and proliferated to produce various degrees of confluency. After exposure to test solutions of polymers, solvents and formulation blends, microscopic examinations were undertaken to establish changes, if any, to the growth and morphology of the cells. In response to a toxic effect, cells become rounded with a granular appearance and detach from the surface of the flask.

### 3.2. Effect of Polymer Dispersions on MAC-T Cells

**Figure 3:**
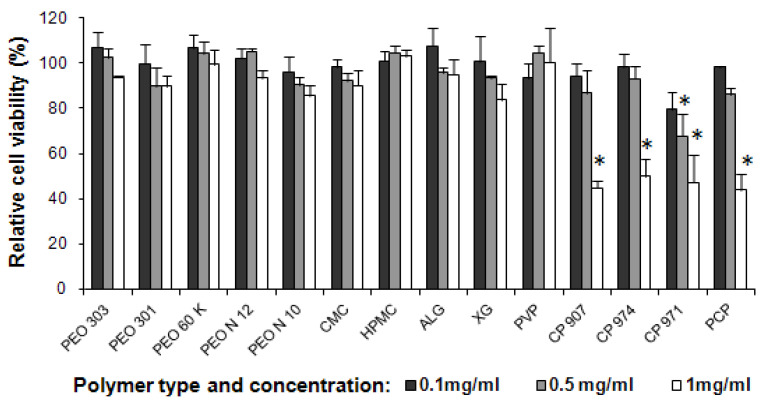
MAC-T cell viability in various concentrations of different polymers after 72 h. Black (0.1 mg/mL), Grey (0.5 mg/mL) and white (1 mg/mL) bars depict the concentration of each polymer. Cell viability was detected by MTT assay (mean ± SD, *n* = 3) and expressed as a percentage of cell viability in untreated control cells. The standard deviation is shown by the error bars. * denotes significance from negative control. (Legend: polyethylene oxide (PEO): MW 7 M (PEO 303), MW 4 M (PEO 301), MW 2 M (PEO 60 K), MW 1 M (PEO N 12), MW 100,000 (PEO N 10), hydroxypropylmethyl cellulose (HPMC), carboxymethyl cellulose sodium (CMC), sodium alginate (ALG), xanthan gum (XG), polyvinyl pyrrolidone (PVP) and carbopols (CP 907, CP 974P, CP 971P) polycarbophil (PCP).

The mitochondrial metabolic activity of viable MAC-T cells in the presence of polymers at three different concentrations ranging from 0.1 to 1 mg/mL was determined using the MTT assay. The cell viability as a function of polymer type and concentration is shown in Figure 3.

PEO, HPMC, CMC, ALG and PVP solutions at the highest concentration of 1 mg/mL showed no significant reduction in cell viability compared to the negative control (Figure 3). XG, at this concentration reduced cell viability to approximately 83% of control, but this was statistically insignificant (*p* > 0.05). In contrast, cell viability decreased significantly (*p* < 0.05) to about 45% relative to the negative control for CP and PCP polymers at 1 mg/mL concentration.

At a lower concentration of 0.5 mg/mL, the cell viability following incubation with PEO, CMC, HPMC, ALG, XG, PVP, CP 907, CP 974 and PCP was comparable with the negative control. On the other hand, CP 971 at this concentration induced a significant (*p* < 0.05) reduction in cell viability to ~68% of control.

At the lowest concentration of 0.1 mg/mL, all polymers, except CP 971 (*p* < 0.05), showed no significant influence on cell viability compared to the negative control.

Of the polymers tested across the specified concentration ranges, only HPMC and PVP had no effect on cell viability as determined by the MTT assay (Figure 3). All other polymers demonstrated a response that was dependent on the polymer concentration to varying degrees.

PEOs of varying molecular weights, CMC, ALG and XG produced relatively small, statistically insignificant (*p* > 0.05) response fluctuations over the concentrations tested while the CPs and PCP produced a much greater cell toxicity to the same tested variations in polymer concentration.

**Figure 4:**
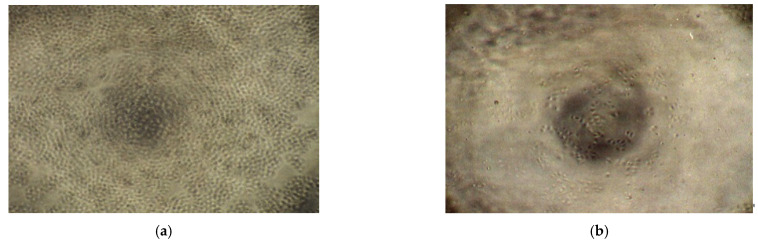
Cell morphology following exposure to polymer solution for 72 h. (**a**) Confluent adherent cells after exposure to HPMC (1 mg/mL) solution and (**b**) reduced cell viability after exposure to CP 971 (1 mg/mL) solution (40× magnification).

Microscopic examination revealed that cells of the negative control wells adhered to the surface and proliferated over the incubation period to produce their characteristic morphology and confluence. The same was observed when incubated with HPMC and PVP at concentrations (up to 1 mg/mL) that had no significant effect on cell viability (Figure 4a). On the other hand, after exposure to high and toxic concentrations of CPs or PCP, cells assumed a rounded shape with the appearance of cytoplasmic granules and had detached from the surface of the flask to disrupt the characteristic ‘cobblestone’ punctate morphology (Figure 4b) and floated in the media.

**Figure 5:**
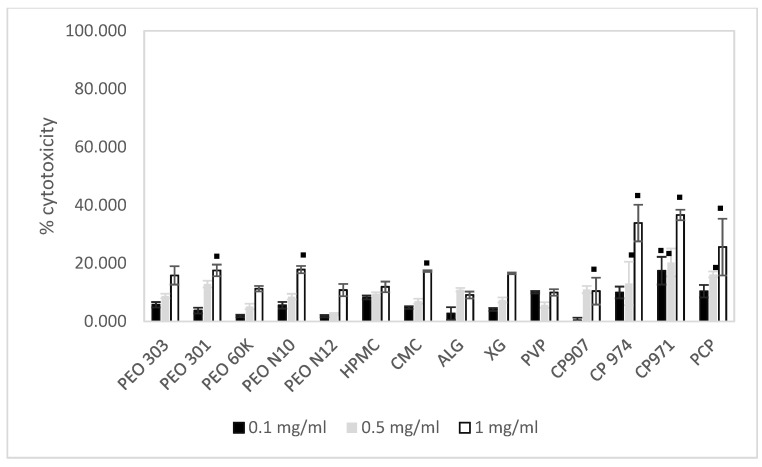
Cytotoxicity of polymer solutions on MAC-T cells after 72 h incubation as detected by the LDH assay (mean ± SD, *n* = 3). Black (0.1 mg/mL), grey (0.5 mg/mL) and white (1 mg/mL) bars depict the concentration of each polymer. The standard deviation is shown by the error bars. ▪ denotes significance from negative control.

In addition to the MTT assay, the lactate dehydrogenase (LDH) assay was also performed as a measure of enzyme activity in the supernatant. The cytotoxicity of different concentrations of the various polymers on MAC-T cells following 72 h incubation is shown in Figure 5.

In most cases, a concentration-dependent cytotoxic effect was observed following incubation, depending on the polymer used. The level of toxicity for all polymers, except PVP, increased with increasing polymer concentrations.

At the highest concentration of 1 mg/mL, all polymers showed a toxic effect on cells as measured by the LDH assay, although enzyme release caused by HPMC, ALG and PVP (9–12%) was significantly lower (*p* < 0.05) than PEO 303, PEO 301, PEO N 10, CMC and XG (16–18%). Significantly higher LDH release (26–37%) was demonstrated by CP974, CP971 and PCP at 1 mg/mL relative to all other polymers (*p* < 0.05) following exposure to MAC-T cells for 72 h.

### 3.3. Effect of Co-Solvents on MAC-T Cells Viability

**Figure 6:**
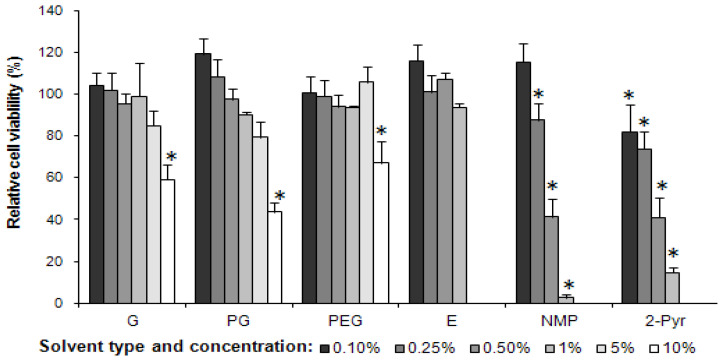
MAC-T cell viability in various concentrations of different solvents after 72 h. Black, various grades of grey and white bars depict the concentration of each solvent ranging from 0.1, 0.25, 0.5, 1, 5 and 10%. Cell viability was detected by MTT assay and expressed as a percentage of viability of untreated control cells (mean ± SD, *n* = 3). The standard deviation is shown by the error bars. * denotes significance from negative control. (Legend: glycerin (G), propylene glycol (PG), polyethylene glycol 400 (PEG), ethanol (E), N-methyl-2-pyrrolidone (NMP) and 2-pyrrolidone (2-Pyr).

The cytotoxicity of co-solvents used in the study to MAC-T cells was determined by the MTT assay following 72 h incubation. The effect of the different concentrations of the various solvents on cell viability is presented in Figure 6. Solvents, G, PG and PEG were tested at concentrations ranging from 0.1–10% *v/v* while E, NMP and 2-Pyr were tested at concentrations ranging from 0.1–1% *v*/*v*. (Results for NMP and 2-Pyr at concentrations above 1% are not shown due to negligible cell viability at these concentrations).

All solvents tested showed a decreasing cell viability with increasing solvent concentration. At the highest concentration of 10% *v*/*v*, G, PG and PEG reduced the cell viability significantly (*p* < 0.05). PG caused the greatest reduction in viable cells followed by G and then by PEG with cell viability being ~45, 60 and 67%, respectively. Decreasing the solvent concentration of all three solvents showed an accompanying increase in cell viability relative to control.

Ethanol tested at concentrations increasing from 0.1 to 1% did not significantly decrease cell viability, while NMP and 2-Pyr demonstrated a concentration-dependent cell viability. At all concentrations of 2-Pyr and at concentrations above 0.25% of NMP, cell viability was adversely affected compared to untreated control samples (*p* < 0.05).

**Figure 7:**
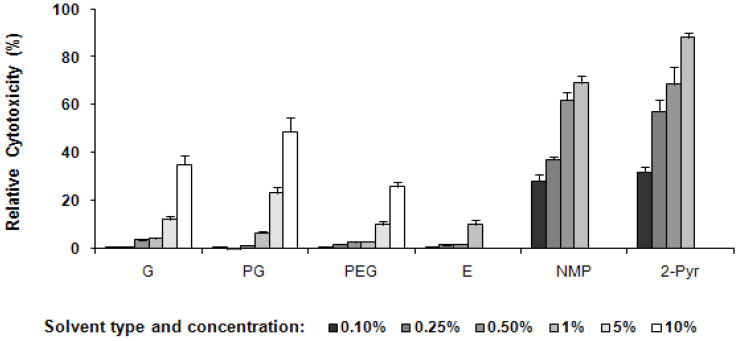
Cytotoxicity of co-solvents on MAC-T cells after 72 h incubation as detected by the LDH assay ((mean ± SD, *n* = 3). Black, various shades of grey and white bars depict the concentrations of each solvent system ranging from 0.1, 0.25, 0.5, 1, 5 and 10%. The standard deviation is shown by the error bars.

In the LDH assay, a similar concentration-dependent cytotoxic effect was observed for the solvents after a 72 h incubation period with MAC-T cells (Figure 7). PG appeared to be more toxic compared to G and PEG. The LDH release due to PG at 10% concentration was ~50% compared to 33% for G and 22% for PEG. On the other hand, 2-Pyr and NMP were highly toxic at 1% concentration where the membrane damage and enzyme leakage caused by 2-Pyr was over 80% and ~65% from NMP. Ethanol did not show any significant toxicity in MAC-T cells at the concentrations used in this study.

It is worthwhile pointing out that our selection of proper solvents for further formulation development was mainly based on the MTT/MTS results as this assay was deemed to be more differentiating and sensitive to concentration changes (Figure 7).

Nevertheless, both the MTT and LDH assay results show relatively good correlation for the various solvents at the concentrations used.

### 3.4. Effect of Polymer/Solvent Formulations on Viability of MAC-T Cells

**Figure 8:**
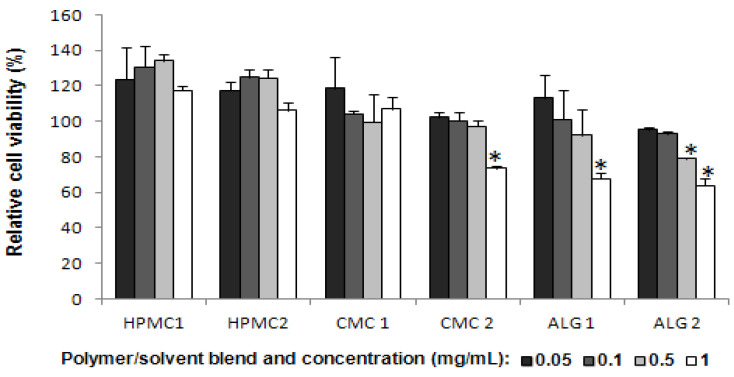
MAC-T cell viability in various concentrations of different formulated polymer/solvent sol phase blends after 72 h. Black, dark grey, light grey and white bars depict the various concentrations of each blend: 0.05 mg/mL, 0.1 mg/mL, 0.5 mg/mL, 1 mg/mL. Cell viability was detected by MTT assay and eexpressed as a percentage of cell viability in untreated controls (mean ± SD, *n* = 3). The standard deviation is shown by the error bars. * denotes significance from negative control. (Legend: HPMC1 = HPMC 10%, G 49.5%, E 22.5%, W 18%; HPMC2 = HPMC 10%, G 9%, PG 72%, W 9%; CMC 1 = CMC 10%, G 18%, E 45%, W 27%; CMC 2 = CMC 10%, G 27%, E 45%, W 18%; ALG 1 = ALG 10%, G 31.5%, PG 31.5%, W 27%; ALG 2 = ALG 10%, G 18%, PG 36%, W 36%; W = water as co-solvent).

Six blends, HPMC1, HPMC2, CMC1, CMC2, ALG1 and ALG2 formulated according to compositions shown in Table 1, were diluted with culture media to concentrations ranging from 0.05 to 1 mg/mL. The viability of MAC-T cells, following exposure to these varying concentrations of polymer-solvent blends, was assessed using the MTT assay; the results are presented in Figure 8.

HPMC and CMC formulations at concentrations of up to 0.5 mg/mL did not exhibit any reduction in MAC-T cell viability following 72 h, on the contrary, cells seem to proliferate in their presence, although this observation should be taken with caution (Figure 8). At a higher concentration of 1 mg/mL of HPMC 1, HPMC2 or CMC1, cell viability was not compromised relative to untreated controls. However, CMC2 at this concentration significantly decreased cell viability to about 74% (*p* < 0.05).

A decreasing trend in cell viability was observed upon exposure of the MAC-T cells to increasing concentrations of ALG 1 and ALG 2 formulations, with significant reductions seen at 1 mg/mL for both formulations and at 0.5 mg/mL for ALG2.

The addition of water (Table 1) to the polymer/solvent mixture is pivotal to induce the desirable sol-to-gel phase change and the subsequent emergence of previously described microstructures [22]. Consequently, the biocompatibility of the formulations was evaluated. Results obtained with the MTT assay showed that formulations HPMC 1, HPMC 2 and CMC 1 did not reduce MAC-T cell viability up to a concentration of 1 mg/mL. On the other hand, a concentration-dependent reduction in cell viability was demonstrated by CMC 2, ALG 1 and ALG 2. At 1 mg/mL concentration a significant reduction in cell viability was produced by these formulations (Figure 8). At a concentration of 0.5 mg/mL, cell viability was not affected by any of the formulations except ALG 2.

### 3.5. Macroscopic Examination of In Situ Gelled Polymer/Solvent Blends in Excised Bovine Teats

Following the infusion of each polymer/solvent blend (formulations HPMC 1, HPMC 2, CMC 1, CMC 2, ALG 1 and ALG 2) into excised bovine teats for the specified times (3 and 12 h) at room temperature under conditions of shaking at 50 rpm, teats were cut open and evaluated for evidence of in situ gelling of the blend formulations. In addition, macroscopic observations of the condition of the teat and the mucosal tissue in direct contact with the gelled formulation were made. In particular, the internal mucosal surface of the teat was scrutinised for any untoward event (colour change, redness, necrosis, inflammation, excess vascularisation etc.).

Three bovine teats, cut open prior to infusion of any polymer/solvent blends, revealed healthy looking mucosal lining with firm, turgid tissue, of vibrant colour with no signs of inflammation or visual evidence of perfuse vascularisation. Although these studies were qualitative in nature, they clearly showed that all six formulations underwent in situ phase transitions inside the teat canal after 3 and 12 h. All formulations were well retained and assumed the shape of the teat cavity lying in close contact to the internal mucosa. There was no evidence of formulation leakage from the teat orifice during the course of the experiment.

**Figure 9:**
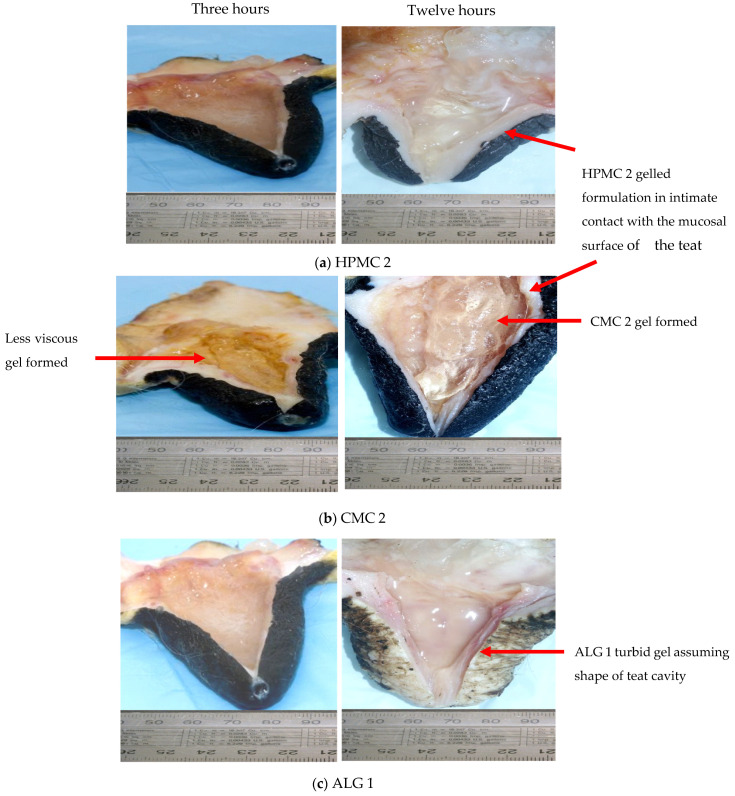
Representative photographs of the internal teat demonstrating in situ formed gels of (**a**) HPMC 2 (**b**) CMC 2 (**c**) ALG 1 after exposure to the infused formulations for 3 and 12 h. (HPMC1 = HPMC 10%, G 49.5%, E 22.5%, W 18%; HPMC2 = HPMC 10%, G 9%, PG 72%, W 9%; CMC 1 = CMC 10%, G 18%, E 45%, W 27%; CMC 2 = CMC 10%, G 27%, E 45%, W 18%; ALG 1 = ALG 10%, G 31.5%, PG 31.5%, W 27%; ALG 2 = ALG 10%, G 18%, PG 36%, W 36%; W = water as co-solvent).

For all formulations, gels formed after three hours were less viscous and had a runny consistency compared with those at twelve hours. In addition, there was no evidence of gels reverting to the sol state with time as intact gels of all formulations were still visible even after 12 h (Figure 9).

A comparison of HPMC, CMC and ALG formulations revealed that gels formed from formulations comprising cellulose derivatives were more viscous and firmly set compared to those formulated using ALG. In addition, these gels were transparent and clear, indicating a homogeneous mixture of excipient and solvents. The gels formed from the two ALG 1 and ALG 2 suspensions were similar in consistency and appeared to be the least viscous compared with those based on CMC and HPMC. At twelve hours, ALG gels, although set, appeared turbid, indicating possible microbial growth or phase separation. Nevertheless, both ALG 1 and ALG 2 appeared firmer at twelve hours than at three hours, where they assumed shape of the teat cistern.

A comparison of the HPMC formulations revealed that the gel formed in situ from HPMC 2 appeared to be more viscous compared to HPMC 1. Among the two CMC formulations, gel consistency was quite similar in both systems by visual inspection. The time, high costs and milk contamination risks associated with an in vivo study render such an ex vivo testing with excised teats attractive for proof-of-concept retention and biocompatibility studies. Macroscopic analysis of the teats after 3 and 12 h of exposure to the gelled formulations revealed no untoward event. The internal tissue of the teat appeared of normal colour, with no visible vascularisation, looked healthy and turgid with no evidence of inflammation compared to untreated control teats. The higher toxicity of polymer blends CMC2, ALG1 and ALG2 toward MAC-T cells seen in the MTT assay (Figure 8) does not correlate with the healthy state of the teat according to macroscopic observations.

## 4. Discussion

Intramammary formulations that are used to prevent bovine mastitis would usually require direct and intimate contact with the epithelial cells of the teat and cistern for prolonged periods of time spanning the dry and drying off periods. Biocompatibility is considered an essential requirement for such dosage forms. Biocompatibility is described as the ability of a product to be used for a specific application without having toxic or deleterious effects to the intended local environment and to biological function [30,31]. Furthermore, assessment of the cytotoxic potential of individual materials used in its manufacture is required to minimise any potential harm to the animal while ensuring the appropriateness of the product for its intended purpose.

Previous studies have shown that MAC-T cells mimic primary mammary epithelial cells (MEC) and would therefore be a valid alternative to MEC [28]. MAC-T cells require a population doubling time of approximately 17 h and have undergone more than 350 passages without signs of senescence [32,33].

Further, this cell line was sufficiently sensitive for two of the commonly used direct-contact methods, the MTT and the LDH assays, for analysing the effects on cell growth inhibition and/or cell death. The 3-(4,5-dimethyl-2-yl)-5-(3-carboxymethoxyphenyl)-2-(4-sulfophenyl)-2H-tetrazolium (MTT) is a standard colourimetric assay for measuring cellular proliferation and viability in the screening of individual solvents and polymeric materials as potential candidates for formulation. The assay measures of mitochondrial bioreduction of a tetrazolium salt to a coloured formazan product by mitochondrial dehydrogenases in living but not in dead cells [34]. The quantity of the formazan product is directly proportional to the number of living cells in culture. In this way, the MTT can detect reversible functional cell damage. On the other hand LDH assay detects the irreversible cell damage [35]. Lactate dehydrogenase (LDH), a stable cytoplasmic enzyme, catalyses the reversible reaction between pyruvic and lactic acids present in all living cells. This enzyme is present in nearly all types of metabolising cells. It rapidly releases into the cell culture supernatant upon cell death or membrane damage and can be measured quantitatively to provide an accurate measure of cell viability and cell membrane integrity [15,36,37]. The long-term incubation time of three days used in this study allowed contact of the test substance with the cells over several cell cycles (~3 cycles), including during their exponential growth phase. Substances that are toxic are likely to induce membrane damage and impairment of the metabolic activity in the cell, which may finally lead to cell lysis and death.

Our MTT assay results clearly showed that the mitochondrial metabolic activity of MAC-T cells was not significantly affected by PEO, HPMC, CMC, ALG and XG at a concentration of 1 mg/mL for up to three days of exposure. The cell viability was always greater than 80%. No changes in cell morphology were observed when examined under a light microscope. In particular, HPMC did not reduce the cell viability of MAC-T cells at any concentration used in this study and the response was comparable with that of the control. Likewise, CMC did not significantly reduce MAC-T cell viability at a concentration up to 1 mg/mL for an incubation period of three days, suggesting that CMC is devoid of cytotoxic effects

ALG and XG also showed very good biocompatibility in both MTT and LDH assays. The % cell viability was always more than 80% following a 72 h incubation period determined by the MTT assay. LDH assay and microscopic observation showed these polymers to be non-toxic to MAC-T cells. Previous studies performed in different cell lines have demonstrated their low toxicity and biocompatibility [38,39,40,41]. However different studies reported varying results related to toxicity of ALG. These could be associated with the presence of impurities or the differences in the source of polymers. Paul de Vos [42] reported that the biocompatibility of a ALG-PLL microcapsule was dependent on the content of the guluronic acid group. Higher biocompatibility was demonstrated by lower guluronic acid content microcapsules implanted in the peritoneal cavity of an AO (antiorthostatic) rat. Similarly, it has been suggested that the cytotoxic effect observed from ALG gel dressing Kaltostat is due to Ca^2+^ release [40]. These results shed light on cell polymer interactions with emphasis on the role of the physicochemical properties of these polymers on their biocompatibility and efficacy for mastitis treatment [43].

Amongst the PEOs of different molecular weight, PEO N 10 had the lowest molecular weight (MW 100K). While it appeared to reduce cell viability in a concentration-dependent manner, i.e., by 18% at a concentration of 1 mg/mL, 9% at 0.5 mg/mL and 6% at 0.1 mg/mL (Figure 2), such reduction was not significant. Previous studies have reported that PEO cytotoxicity increases with decreasing molecular weight [44,45,46]

Carbopols and PCP showed significantly higher toxicity in both assays. A concentration-dependent cytotoxic effect was observed from CPs and PCP. CPs and PCP at 1 mg/mL concentration reduced cell viability to below 50%. These results were in agreement with the results reported in the literature by Adriaens et al. [47], who observed increased mucus secretion and LDH release with a spray-dried Amoica/carbopol 974 mixture in a mucosal irritation study of slug mucosa. The membrane damage was more pronounced with a higher concentration of carbopol in the formulation. Similar results were reported by Diebold [48] in SIRC cells (rabbit corneal cells) and Debbasch (2000) in human conjunctive cells, who observed severe toxic effects caused by commercial carbomer gel formulation after only a 30 min exposure period [49].

CP 971 caused the highest toxicity to MAC-T cells based on our results for both assays. Furthermore, CP 971 was also found to be the most toxic of all polymers tested, followed by PCP. These results agree with the results reported by Ugwoke et al. [50], who found CP 971 to be very toxic to human nasal primary cell culture in vitro and to rabbit nasal mucosa in vivo. They concluded that this polymer was an inappropriate vehicle for delivery to the nasal mucosa [50]. CPs are anionic, cross-linked polyacrylic acid polymers with high viscosity. It has been reported that CPs and PCP are utilised to increase membrane permeability and hence act as penetration enhancers in formulations. However, the enhanced permeability could be due to the disruption of the tight junction that increases paracellular permeability [51,52]. The increased cellular permeability resulting from the disruption of the cell membrane causes leakage of the intracellular enzyme LDH into the extracellular fluid [53]. The results of our study demonstrated this. Based on the toxicity profiles obtained in our study, CP and PCP polymers were not progressed further for use in formulation development.

The different physicochemical properties of polymers, such as hydrophobicity, hydrophilicity, distribution of charge, crystallinity, average molecular weight and residual monomer, may contribute and invoke different responses in cells [54]. Considering the complex interplay between the physicochemical properties and cytotoxic effect of these polymers on membrane integrity, it is difficult to interpret these results because of the limited number of materials tested. In general, the toxicity of organic compounds increases as molecular weight decreases. This is because the solubility and tissue diffusion of such compounds increases as the molecular weight decreases [55].

All solvents used in this study demonstrated a concentration-dependent cytotoxic effect. Organic solvents are known to cause an increase in the disrupted area of the intracellular membrane, causing LDH release and eventual cell death.

Among the solvents investigated in a 0.1–10% concentration range, PEG demonstrated the highest biocompatibility in both assays compared to G and PG. Total biocompatibility up to 5% PEG was observed with a decline when the concentration was increased to 10%. However, this cytotoxicity was significantly reduced compared to G and PG in both assays at this concentration. PG caused the highest cell death at 5 and 10% concentrations. At these higher concentrations of solvents, visible signs of cell rounding along with the expansion of intercellular spaces were observed.

Among the solvents tested in a 0.1–1% concentration range, E was the most biocompatible, while NMP and 2-Pyr caused higher toxicity to MAC-T cells as demonstrated in both assays. Microscopic observations indicated that the cell deaths occurred as early as 3 h after the addition of these solvents. At concentrations greater than 0.25% NMP and greater than 0.1% 2-Pyr, a marked drop in cell viability was seen. Toxicity with NMP was also endorsed in a study with rats exposed to 1 mg/L NMP. The animals, while showing no significant clinical signs at lower concentrations (0.1 and 0.5 mg/L), were lethargic, had respiratory difficulty and had shown excessive mortality while at 1 mg/mL [56].

It has been reported that the rank order for release of creatine kinase enzyme from isolated rat muscles following injection of 40% *v/v* solution was PG > E > PEG [57]. In another study, PEG caused lower death rate of cultured endothelial cells (HUV-EC) compared to E [58].

The data obtained for the MTT and LDH for the polymers and solvents tested showed good correlation (results not shown), which further validated the results. Based on the biocompatibility profiles, the polymers HPMC, CMC and ALG, as well as solvents G, PG and E, were selected for further development of in situ gel formulations for intermammary administration. The polymer–solvents sol blends formed in situ gels following administration to excised teats in conditions simulating the natural teat environment. The formation of a different microstructure (of the gel) in situ, and the presence of co-solvents as part of the formulation may influence cell viability. Consequently, the cytotoxicity of the formulations over a 0.05–1 mg/mL concentration range was evaluated. Results determined by MTT assay showed that formulations HPMC 1, HPMC 2 and CMC 1 had no effect on MAC-T cell viability up to the highest concentration tested. On the other hand, a concentration-dependent reduction in cell viability was observed with CMC 2, ALG 1 and ALG 2. At 1 mg/mL of blend, a significant reduction in cell viability was induced by these formulations (Figure 7). At a concentration of 0.5 mg/mL, cell viability was not affected by any of the formulations except ALG 2 which contained 10% alginate, 18% G, 36% PG and 36% water. The results seen with formulation ALG2 may be attributable to the concentrations of the solvents and co-solvent, contamination or a combination of these. The toxicity of the solvent PG at 5 and 10% was shown previously in Figure 6. Both G and the high concentrations of water in this formulation may have increased the sensitivity of the MAC-T cells and contributed to hypotonic stress conditions created over the prolonged incubation period, hence inducing cell lysis. Selzner et al. [59] demonstrated that exposure of cells to distilled water for more than 15 min was associated with significant increases in LDH levels and cell lysis.

The mammary gland of a cow is very sensitive and susceptible to provocation by the introduction of foreign materials, which, if harmful, is likely to manifest as an inflammatory event. Our ex vivo teat model took advantage of this to visualise any visual signs of an inflammatory effects of the in-situ gelling formulations to the teat tissue over the period of study.

Retention studies in excised mammary glands demonstrated that all formulations were gelled in three hours. All the formulations were well retained and demonstrated macroscopic biocompatibility with no signs of local irritation or inflammation inside the teat canal while agitated at 50 RPM for the experimental period of up to 12 h. There was no evidence of inflammation, hyperaemia, clotting, redness or necrosis. These observations further supported the in vitro cell viability testing. These formulations could potentially be of value as insertable/implantable mucoadhesive systems for sustained intramammary drug delivery [60,61].

## 5. Conclusions

In this study, the effect of different hydrophilic polymers and solvents on MAC-T bovine mammary epithelial cells was investigated. Two assays quantifying the influence of polymers and solvents on the metabolic activity and the membrane integrity of the cells were employed. The results indicated that, except for CPs and PCP, all other polymers studied were biocompatible, as shown by both assays.

Solvents studied induced concentration-dependent cytotoxicity to MAC-T cells. Amongst those studied, 2-Pyr followed by NMP were the most toxic; PEG followed by G, PG and E were the least toxic to MAC-T cells.

In situ gelling formulations HPMC1, HPMC2 and CMC2 blends showed no signs of local undesirable effects using the ex vivo teat model. These in situ gel-forming formulations appeared to be promising as internal teat seals for the prevention of bovine mastitis over the dry and drying off periods. Further, they could provide a platform to develop sustained release intramammary drug delivery systems.

The approach adopting biocompatibility and cytotoxicity studies alongside formulation development enabled screening and selection of candidate materials at an early stage of intramammary dosage form development.

## Figures and Tables

**Table 1:** Composition of polymer/solvent intramammary formulations.

	Polymer/Solvent Blend Composition (% *w/w*)						
	Polymer			Solvent			
	HPMC	CMC	ALG	G	E	PG	W
HPMC 1	10			49.5	22.5		18
HPMC 2	10			9		72	9
CMC 1		10		18	45		27
CMC 2		10		27	45		18
ALG 1			10	31.5		31.5	27
ALG 2			10	18		36	36

Key: HPMC—hydroxypropylmethyl cellulose, CMC—carboxymethyl cellulose sodium, ALG—sodium alginate, G—glycerin, E—ethanol, PG—propylene glycol, W—water.

## Data Availability

Not applicable.
